# Craniofacial speargun injuries: report of three cases, literature review and proposed management guidelines for maxillo-facial surgeons

**DOI:** 10.1007/s10006-026-01534-8

**Published:** 2026-03-11

**Authors:** Luigi Angelo Vaira, Olindo Massarelli, Andrea Biglio, Giovanni Salzano, Antonino Maniaci, Jerome R. Lechien, Giacomo De Riu

**Affiliations:** 1 Maxillofacial Surgery Unit, Department of Medicine, Surgery and Pharmacy, University of Sassari, Sassari, Italy; 2 Maxillofacial Surgery Operative Unit, Department of Medical Biotechnology, University Hospital of Siena, Siena, Italy; 3 Head and Neck Section, Department of Neurosciences, Reproductive and Odontostomatological Science, Federico II University of Naples, Naples, Italy; 4 Department of Medicine and Surgery, University of Enna Kore, Enna, Italy; 5 Department of Surgery, Mons School of Medicine, UMONS, Research Institute for Health Sciences and Technology, University of Mons (UMons), Mons, Belgium; 6 Department of Otolaryngology-Head Neck Surgery, Elsan Polyclinic of Poitiers, Poitiers, France; 7 Department of Medicine, Surgery and Pharmacy, University of Sassari, Viale San Pietro 43/B, Sassari, Italy

**Keywords:** Speargun injury, Craniofacial trauma, Penetrating head injury, Skull base reconstruction, Maxillofacial surgery, Case series

## Abstract

Speargun injuries of the head and neck are extremely rare but potentially devastating because of the high density of vital structures in this region. Most available data derive from isolated case reports, and guidance specific to oral and maxillofacial surgeons remains limited. We report three patients with craniofacial speargun injuries managed in a maxillofacial surgery setting, illustrating three distinct scenarios with increasing complexity: an isolated sinonasal trajectory confined to the midface, an anterior cranial fossa trajectory following a submental/oral entry, and a transoral posterior fossa trajectory associated with diffuse haemorrhage and delayed neurological deterioration. Review of the literature confirms recurrent patterns in entry sites, trajectories, complications and outcomes, and supports several key principles: early airway control, systematic cross-sectional and vascular imaging, multidisciplinary planning, and anatomically driven choice between retrograde and anterograde extraction with watertight skull-base reconstruction. These concepts are crucial for maxillofacial surgeons facing these rare but high-risk injuries.

**Supplementary Information:**

The online version contains supplementary material available at 10.1007/s10006-026-01534-8.

## Introduction

Penetrating injuries of the maxillofacial region are uncommon in civilian practice and are most frequently caused by firearms or stab wounds. In contrast, injuries produced by spearguns or fishing harpoons are exceedingly rare and represent an unusual mechanism of craniofacial trauma [1–3]. These weapons are widely used for recreational spearfishing and water sports, and their projectiles are typically equipped with barbed or flapper tips that are designed to expand or resist retrograde movement, a feature that complicates both the pattern of injury and surgical removal [2, 4]. 

Because of the high density of vital structures within the head and neck, speargun injuries in this region may be life-threatening even when the external wound appears deceptively small. Published cases include isolated maxillofacial injuries without intracranial involvement, often managed by oral and maxillofacial surgeons [1–4], as well as penetrating craniocerebral lesions reported mainly in the neurosurgical literature [5–8]. 

Despite the potential severity of these lesions, the literature consists almost exclusively of single case reports and small series [1–9]. An up-to-date review of facial speargun injuries identified only 13 cases over a 36-year period [4]. 

From a maxillofacial surgery perspective, these injuries raise specific diagnostic and therapeutic challenges. However, current recommendations are extrapolated from a heterogeneous collection of isolated reports, and OMFS-focused data on craniofacial entry patterns, skull-base involvement, reconstructive strategies and long-term functional and aesthetic outcomes remain limited [4, 10]. In this article, we present three patients with head and neck speargun injuries managed in a maxillofacial surgery setting and discuss their management in the context of a targeted review of the literature on craniofacial and transcranial speargun trauma. Our aim is to highlight recurring anatomical patterns and propose practical principles to support decision-making for oral and maxillofacial surgeons confronted with these rare but potentially devastating injuries.

## Case reports

### Case 1

A 23-year-old woman was shot in the face with a speargun by her partner during an assault. On arrival at the emergency department, she was conscious, hemodynamically stable, and complained mainly of facial pain and epistaxis. There was an entry wound in the left paranasal region, with the shaft of the spear protruding externally.

**Fig. 1:**
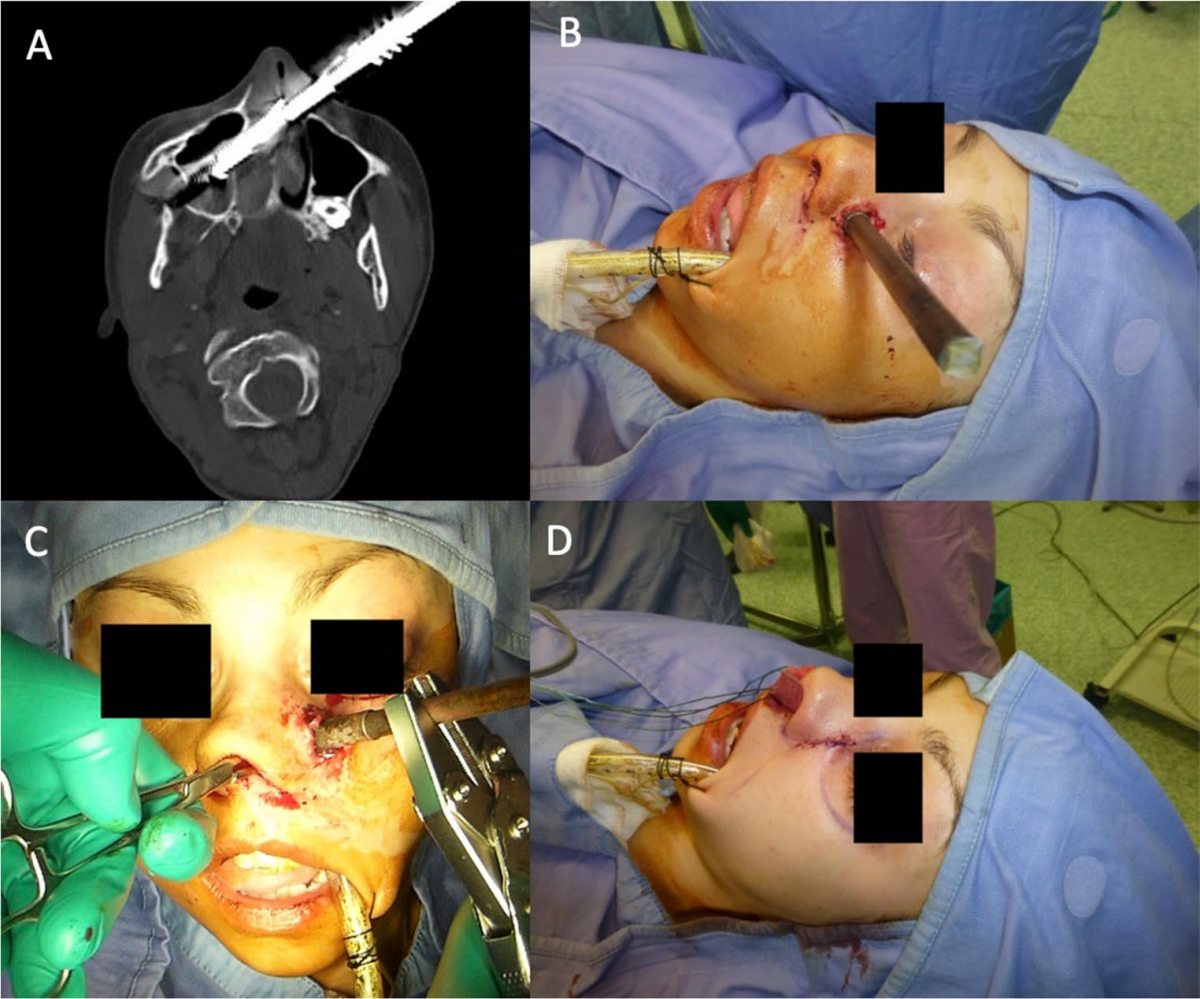
Case 1 – craniofacial speargun injury. (**A**) Axial CT scan showing the spear entering the left paralateral nasal region, crossing both nasal cavities and lodging in the medial wall of the right maxillary sinus. (**B**) Preoperative clinical view with the shaft protruding from the left paranasal area. (**C**) Intraoperative view of controlled retrograde extraction, with a Kocher clamp applied to the barb. (**D**) Immediate postoperative appearance after layered wound closure and bilateral anterior nasal packing

Maxillofacial CT scan (Fig. 1A) showed that the spear entered through the left paralateral nasal region, traversed the left nasal cavity, perforated the nasal septum, crossed the right nasal cavity and penetrated the medial wall of the right maxillary sinus, where the tip was lodged within the sinus. The spear was equipped with a single barb, which was located immediately beneath the cutaneous plane at the entry site. No intracranial, orbital or major vascular involvement was identified.

The patient was taken urgently to the operating room (Fig. 1B). Under general anaesthesia, the spear was removed in a controlled retrograde fashion. A Kocher clamp was applied to the barb and maintained in the closed position throughout extraction to prevent uncontrolled deployment and additional soft-tissue damage (Fig. 1C). After removal of the spear, the wound was copiously irrigated and the cutaneous tract was closed in layers. Bilateral anterior nasal packing was placed, one pack in each nasal cavity, to control postoperative bleeding (Fig. 1D).

Postoperatively, the patient received antibiotic prophylaxis with amoxicillin–clavulanic acid 1 g three times daily. Tetanus immunisation was already up to date. Nasal packs were removed on postoperative day 3, immediately before discharge.

The postoperative course was uneventful. At clinical follow-up, wound healing was satisfactory and no functional or aesthetic sequelae were observed.

### Case 2

**Fig. 2:**
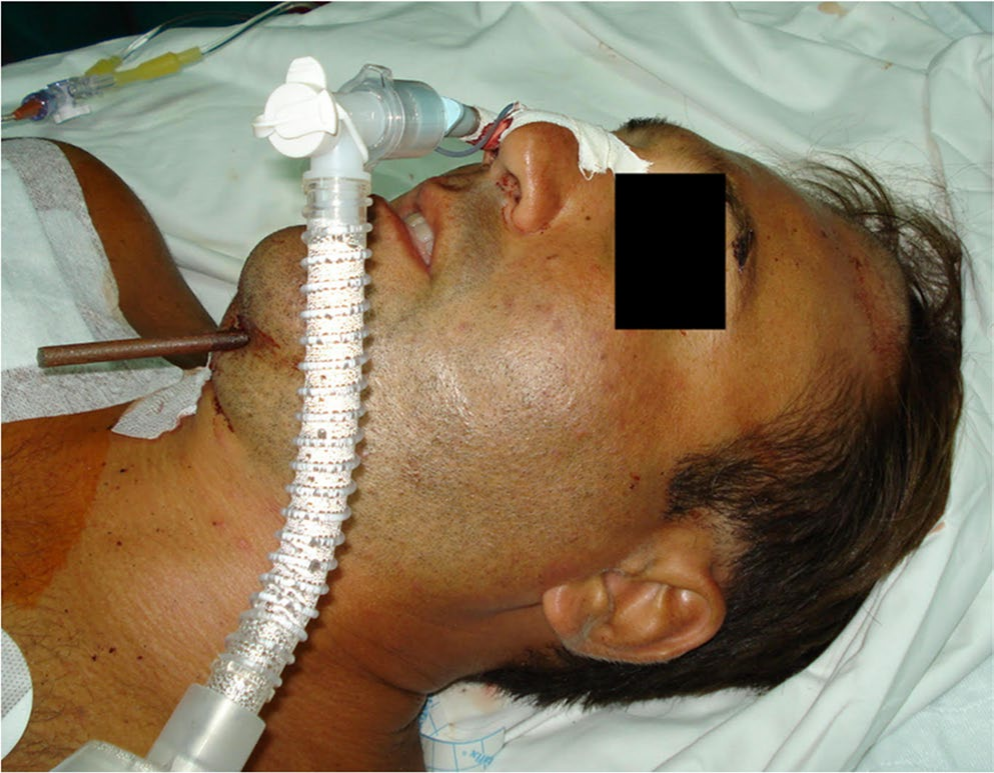
Case 2 – submental speargun suicide attempt. Preoperative clinical view showing the shaft of the spear protruding from the submental region and emerging into the oral cavity

A 37-year-old man attempted suicide by shooting himself with a speargun placed beneath the chin. Emergency medical services found him conscious and hemodynamically stable, with the shaft of the spear protruding from the submental region and emerging into the oral cavity. On arrival at the emergency department, clinical examination revealed an entry wound in the submental area, with the spear traversing the floor of the mouth and perforating the hard palate, the tip occupying the right nasal cavity (Fig. 2). There was no active massive bleeding and no focal neurological deficit.

Because the presence of the shaft within the oral cavity precluded orotracheal intubation and also obstructed the right nasal passage, the patient was sedated and nasotracheally intubated through the left nasal cavity. A maxillofacial and cranial CT scan demonstrated a fishing harpoon entering posterior to the mandibular symphysis. Along its course, the shaft traversed the tongue, perforated the hard palate and extended into the left nasal cavity, then passed through the ipsilateral ethmoid and sphenoid bones to enter the cranial cavity. Intracranially, the spear coursed lateral to the left optic nerve and ascended through the frontal lobe towards the vertex, where it fractured the inner and outer tables of the skull and stopped immediately beneath the scalp. No frank intraparenchymal hematoma or significant intracranial haemorrhage was identified.

The patient was transferred to the operating room for a combined maxillofacial and neurosurgical procedure under general anaesthesia. Given the configuration of the harpoon and the position of the barbed tip, an anterograde removal was deemed safer than retrograde extraction. The shaft was first cut just below the submental entry point to facilitate cranial exposure. A bicoronal incision was performed and a tailored craniotomy was fashioned around the point where the spear emerged from the calvaria, including removal of a small bone operculum surrounding the exit site. Under direct vision, the tip of the harpoon was grasped with a clamp and gently pulled in an anterograde direction until the entire shaft was removed along its original trajectory. The dural defect was then enlarged slightly, thoroughly irrigated and closed with a dural substitute patch reinforced with fibrin glue to achieve a watertight seal. Because of the contamination from the paranasal sinuses and oral cavity, the small bone fragment around the exit site was not repositioned, and the scalp was closed in anatomical layers over the dural repair.

Attention was then turned to the maxillofacial component. The submental entry wound, the laceration of the tongue and the perforation of the hard palate were debrided and closed in layers with resorbable sutures. An anterior nasal pack was placed in the left nasal cavity to control postoperative bleeding. Postoperatively, the patient received broad-spectrum intravenous antibiotic prophylaxis with ceftriaxone 2 g once daily and metronidazole 500 mg three times daily for 7 days, followed by oral amoxicillin–clavulanic acid for an additional 7 days. Antiepileptic prophylaxis with levetiracetam was administered for one week. The nasal pack was removed on postoperative day 3.

Control CT scans at 12 h and on postoperative day 3 confirmed correct positioning of the cranial reconstruction and excluded the development of delayed intracranial haemorrhages or new lesions. The postoperative course was uneventful, with no neurological deficit or signs of infection. The patient was discharged 10 days after the trauma with normal neurological examination and was referred for psychiatric evaluation and ongoing treatment of his depressive disorder.

### Case 3

**Fig. 3:**
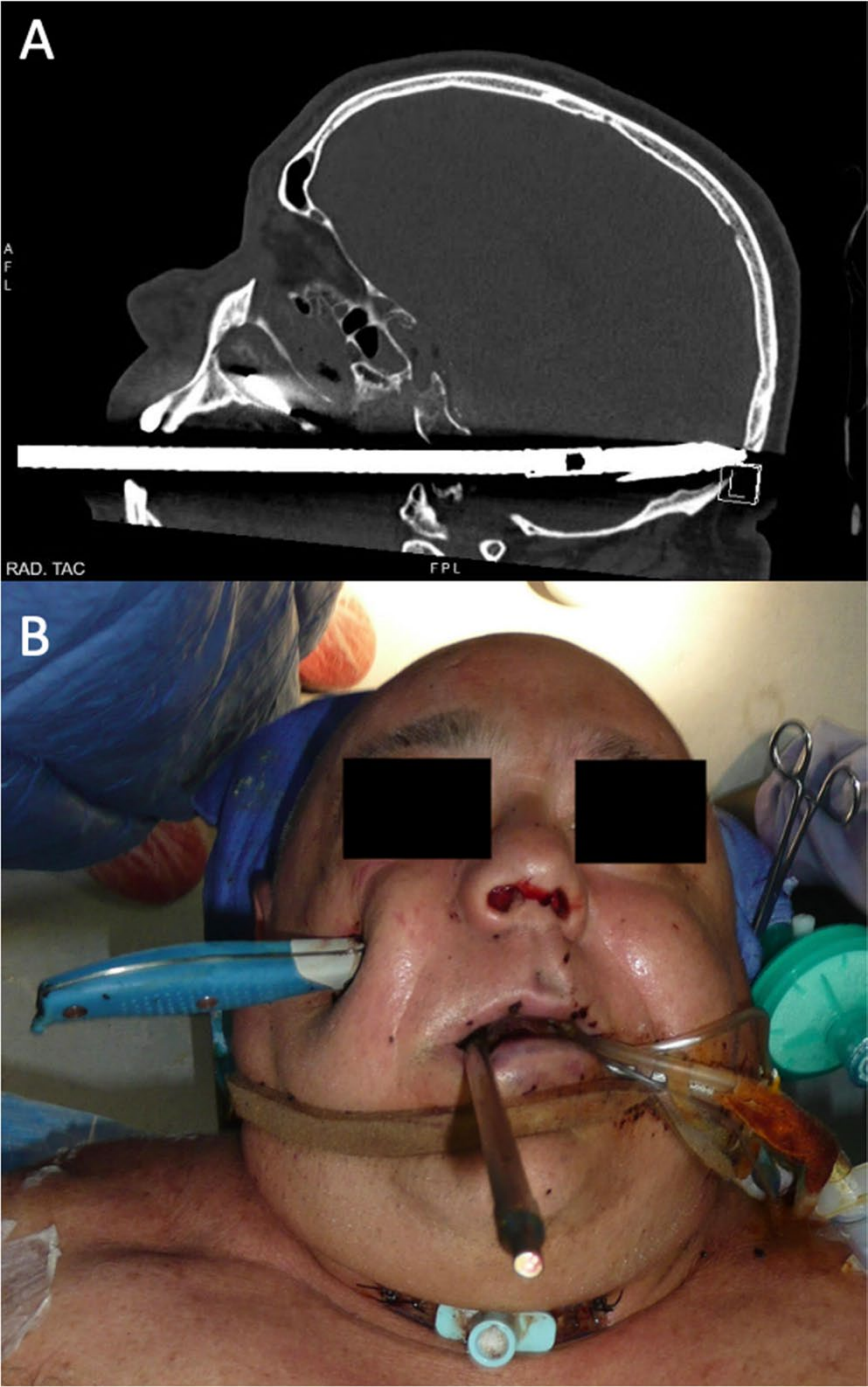
Case 3 – combined transoral speargun and facial stab injury. (**A**) Sagittal CT scan showing the fishing harpoon entering the oral cavity, crossing the skull base just above the atlas, traversing the right cerebellar hemisphere and fracturing the occipital bone, with the tip lodged in the occipital soft tissues. (**B**) Preoperative clinical view with the spear shaft protruding from the mouth and the knife penetrating the right cheek

A 60-year-old man was assaulted by his son, who shot him in the face with a speargun and stabbed him in the right zygomatic region with a kitchen knife. Emergency medical services found the patient unconscious at the scene and he was immediately orotracheally intubated. On arrival at the emergency department, the patient remained sedated, mechanically ventilated and haemodynamically stable. Initial CT scan of cranio-maxillo-facial region demonstrated a fishing harpoon entering the oral cavity and penetrating the skull base just above the level of the atlas in the right paramedian region (Fig. 3). The shaft crossed the clivus and traversed the right cerebellar hemisphere, reaching the occipital bone where it caused a comminuted fracture, with the tip located in the subcutaneous tissues of the occipital scalp. In addition, a knife was seen entering the right cheek, passing obliquely through the right maxillary sinus, perforating the hard palate and crossing the left maxillary sinus to reach the left zygomatic region, where the tip lay in the overlying subcutaneous tissues, associated with a fracture of the left zygoma. Intracranially, there were multiple haemorrhagic foci involving the right cerebellar hemisphere, the interhemispheric falx, the basal cisterns, the sylvian cistern and the parieto-occipital sulci bilaterally, consistent with diffuse subarachnoid haemorrhage.

The patient was transferred emergently to the operating room for combined maxillofacial and neurosurgical management. A temporary tracheostomy was performed to secure the airway and facilitate prolonged ventilatory support. Given the configuration of the harpoon and its intracranial trajectory, an anterograde removal was considered safer than retrograde extraction. The shaft was cut intraorally just anterior to its entry point in the oropharynx to allow posterior access. A midline occipital scalp incision was then made and a craniotomy was fashioned around the point where the tip of the harpoon emerged through the occipital bone, removing a bone flap that incorporated the fracture margins. Under direct vision, the tip of the harpoon was grasped with a heavy clamp and gently pulled in an anterograde direction, following its original trajectory through the cerebellum and clivus until the entire shaft was removed.

The surgical cavity was copiously irrigated. The dural defect in the posterior fossa was carefully inspected, slightly enlarged to allow debridement of devitalised tissue, and then closed using a dural substitute patch reinforced with fibrin glue in order to obtain a watertight closure and minimise the risk of cerebrospinal fluid (CSF) leakage. Because of the extensive contamination from the aerodigestive tract and paranasal sinuses, the occipital bone fragment was not repositioned; the posterior fossa was reconstructed as a craniectomy and the scalp was closed in layers over the dural repair.

Attention was then directed to the midfacial stab wound. Through a combination of intraoral and transcutaneous approaches, the knife was exposed along its entire course. Under direct visual control, it was withdrawn along the entry trajectory to avoid additional damage to the maxillary sinuses, palate and left zygomatic region. The maxillary and palatal lacerations were debrided and closed with resorbable sutures; the zygomatic fracture was stabilised with internal fixation. All wounds were thoroughly irrigated before closure.

Postoperatively, the patient was admitted to the intensive care unit (ICU) for continued sedation and mechanical ventilation. Broad-spectrum intravenous antibiotics were administered, consisting of ceftriaxone 2 g once daily, metronidazole 500 mg three times daily and vancomycin 1 g twice daily for 10 days, to cover oral, sinus and skin flora and to reduce the risk of meningitis or intracranial abscess. Antiepileptic prophylaxis with levetiracetam was given for one week.

An immediate postoperative CT scan demonstrated diffuse subarachnoid haemorrhage, intraventricular blood and multiple haemorrhagic foci in the right cerebellar hemisphere, together with pneumoencephalus in the posterior fossa and supratentorial compartment. Serial CT scans over the first three postoperative days showed a gradual reduction of the subarachnoid haemorrhage, partial resorption of intraventricular blood and regression of pneumoencephalus, but progressive ventricular enlargement was noted, consistent with post-haemorrhagic hydrocephalus. Given the increasing ventricular size and the patient’s persistent impaired level of consciousness when sedation was lightened, a ventriculo-peritoneal shunt was placed, with subsequent stabilisation of ventricular dimensions on follow-up imaging.

The patient remained in the ICU for 43 days because of prolonged neurological impairment and the need for ventilatory and haemodynamic support. Over time, he was weaned from mechanical ventilation and the tracheostomy was decannulated. On transfer to the neurosurgical ward, neurological examination revealed severe right cerebellar syndrome with marked limb ataxia on the right side, dysmetria, truncal instability, gait inability, and scanning dysarthria, in keeping with the MRI findings of extensive persistent damage to the right cerebellar hemisphere. There was also mild dysphagia requiring dietary modification and supervision during feeding, but no major supratentorial cognitive deficit.

Sixty-seven days after the trauma, the patient was transferred to a specialised neurorehabilitation centre for intensive physiotherapy and speech therapy. Despite gradual functional gains, his overall condition remained fragile, and 35 days after admission to the rehabilitation unit he died from pulmonary complications, attributed to severe pneumonia and respiratory failure in the context of prolonged immobilisation and neurological disability.

## Discussion

Speargun injuries involving the head and neck remain exceptionally rare. From a ballistic standpoint, however, even when long metallic shafts penetrate deeply into the brain, these injuries differ substantially from high-energy firearm wounds. Speargun projectiles are relatively low-energy, “cold” objects and do not reproduce the extensive cavitation, thermal damage and intralesional gas expansion associated with modern gunshots, which are more frequent in our societies and are almost invariably fatal. As a consequence, many patients with craniofacial speargun injuries present haemodynamically stable and sometimes with surprisingly preserved neurological function, despite dramatic imaging findings. Nevertheless, delayed complications such as haemorrhage, oedema, hydrocephalus or infection may still lead to devastating neurological sequelae, making the management of these patients particularly delicate. The three cases presented here illustrate the full spectrum of maxillofacial and cranial involvement, ranging from an isolated sinonasal trajectory managed by oral and maxillofacial surgeons alone (Case 1) to two complex transcranial injuries requiring combined maxillofacial and neurosurgical management (Cases 2 and 3). In our review of the literature, we identified 19 published cases of head and neck speargun injuries [Supplementary Table 1] [1–23]. When analysed together with the published literature, our series highlights recurring anatomical patterns, common pitfalls and key principles for safe management.

### Demographics and mechanism of injury

Consistent with previous reports, most published patients are male young or middle-aged adults, with a mixture of accidental, suicidal and assault-related mechanisms [1–4, 6–12, 19–23]. Accidental injuries typically occur during recreational spearfishing or handling the loaded gun and often involve the orbit, frontal region or midface [1–3, 6, 12, 15, 16]. By contrast, suicidal attempts tend to present with submental or intraoral entry and upward cranial trajectories, frequently leading to transcranial penetration [7–10, 17, 18]. Assaults are less commonly reported but may show similar patterns to suicidal injuries, with close-range shots to the face or oral cavity [5, 11, 19, 21, 23]. 

Importantly, the demographics and apparent incidence of speargun-related trauma are likely influenced by the characteristics of the underlying population [24, 25] and its exposure to underwater fishing activities. In our setting (Sardinia, Italy), recreational spearfishing is particularly widespread in a territory with a strong maritime vocation and easy access to the sea, which entails a high local prevalence of speargun use. The fact that we report three craniofacial speargun injuries from a single maxillofacial unit, despite the very small number of cases described worldwide, may therefore reflect these regional patterns of exposure rather than a truly increased intrinsic risk associated with the weapon itself.

Our series mirrors the mechanisms reported in the literature: one interpersonal violence case with limited craniofacial involvement (Case 1), one suicidal attempt via a submental entry with frontal lobe penetration (Case 2), and one assault with a transoral trajectory to the posterior fossa (Case 3). These mechanisms are crucial for initial suspicion of skull-base or intracranial involvement even when external wounds are small.

### Entry sites, trajectories and structures at risk

The entry points and trajectories observed in our patients are typical of those previously described. Alper et al. [1] and Ribeiro et al. [3] reported extracranial trajectories confined to the paranasal sinuses and maxillofacial skeleton, often with the barb lodged in the maxillary sinus or nasal cavity, similar to our first case. Macedo Costa et al. [4] described an isolated mandibular fracture caused by a metallic spear with no intracranial extension. In these cases, management is mainly within the remit of oral and maxillofacial surgery, albeit with careful radiological planning [1–4, 21, 22]. 

In contrast, a large proportion of published cases exhibit intracranial or spinal canal penetration [5–11, 13–18]. Orbital trajectories through the roof into the frontal lobe or anterior cranial fossa have been frequently reported, sometimes with associated vascular injury or traumatic aneurysm [5, 6, 15, 16]. Posterior fossa trajectories, similar to our third case, are less common but have been described in suicidal or accidental injuries entering via the mouth and traversing the clivus towards the cerebellum and occipital bone [7, 12, 14, 17]. Clinical consequences range from limited focal cerebellar damage to devastating diffuse subarachnoid haemorrhage and hydrocephalus, as in our patient and in other series of posterior fossa or deep-seated lesions [7, 11, 13, 17]. 

Our second case reproduces the now well-recognised submental/oral pattern in suicidal attempts, with the spear passing through the floor of the mouth, hard palate, ethmoid and sphenoid to reach the frontal lobe and the vertex, analogous to the trajectories described by other authors [7–10, 18]. This pattern underscores the need for a high index of suspicion for intracranial involvement whenever the entry point is submental or intraoral and the shaft is directed cranially.

### Initial assessment and airway management

The first priority in these patients is airway protection and haemodynamic stabilisation. In extracranial or “low” maxillofacial injuries, conventional orotracheal intubation is often feasible [1–3]. However, in many reported cases, including our second and third patients, the presence of a long shaft traversing the oral cavity severely restricts mouth opening or obstructs the oropharynx, making orotracheal intubation impossible [7–10, 17]. In such scenarios, authors have advocated either nasotracheal intubation through the contralateral nasal cavity, as performed in our second case, or primary tracheostomy, as in our third case and in several intracranial series [7–9, 17, 22]. 

A consistent message from the literature is that blind removal or manipulation of the spear at the scene must be strictly avoided, as uncontrolled traction may convert a tamponading foreign body into catastrophic haemorrhage or exacerbate neural injury [5–8, 11–13]. This principle was respected in all three of our patients, with definitive extraction deferred until full imaging and operative planning were available.

### Role of imaging and vascular assessment

High-resolution CT with multiplanar reconstructions is universally considered the cornerstone of pre-operative assessment, allowing surgeons to define the entry point, trajectory and relationship of the spear to the skull base, paranasal sinuses, orbit and brain [1–4, 6–12, 15–17]. In our series, CT was decisive in demonstrating that Case 1 had a trajectory confined to the nasal cavities and right maxillary sinus, justifying a purely maxillofacial approach, whereas Cases 2 and 3 showed clear intracranial extension and warranted combined neurosurgical management.

Several reports highlight the importance of CT-angiography or conventional digital subtraction angiography to detect vascular injuries, particularly when the projectile crosses major arterial territories such as the carotid or vertebro-basilar systems, or when it traverses the circle of Willis or Sylvian fissure [6–8, 11–13, 15, 16, 18, 19]. Traumatic aneurysm formation, arterial dissection, pseudoaneurysm and delayed haemorrhage have all been described after speargun injuries [6, 13, 15]. In our second case, CT imaging showed the spear running lateral to the optic nerve without evidence of vascular injury, and no delayed haemorrhagic complications were observed on serial CT scans. In the third case, the posterior fossa trajectory produced diffuse subarachnoid and intraventricular haemorrhage; progressive ventricular enlargement necessitated ventriculo-peritoneal shunting, consistent with post-haemorrhagic hydrocephalus patterns described in other subarachnoid-rich injuries [7, 11, 13, 17]. 

### Surgical strategy: anterograde vs. retrograde extraction

The presence of a barbed or flapper tip is the defining technical challenge in speargun injuries. Blind traction risks further parenchymal tearing or skull-base laceration; thus most authors emphasise controlled removal under direct vision, ideally along the original trajectory of the spear [1–9, 11, 12, 15–18]. 

In purely extracranial cases, the barb often lies within the nasal cavity or maxillary sinus and can be neutralised or controlled externally, allowing safe retrograde extraction via the entry wound [1–4, 19–22]. Our first case fits this model: the single barb was located just beneath the skin and could be clamped with a Kocher forceps, enabling a carefully controlled retrograde removal without additional tissue damage.

By contrast, intracranial cases have predominantly been treated with anterograde extraction from the cranial exit or the deeper end of the trajectory [5–9, 11, 13, 15–18]. Several authors describe craniotomies tailored around the calvarial exit or intracranial tip, with removal of the surrounding bone flap and gentle anterograde traction under direct visual control [7, 10, 17, 18]. Our second and third cases followed the same principle: the proximal shaft was shortened at the facial entry point, a craniotomy was performed around the exit site (vertex in Case 2, occipital bone in Case 3), and the barb was grasped intracranially and removed along the original path. This approach minimises uncontrolled tearing of brain tissue and permits immediate inspection and debridement of the tract.

In posterior fossa or orbitocranial injuries, where critical neurovascular structures are densely packed, a multidisciplinary approach is especially important [5–8, 12, 15–17]. The orbitocranial series of Chibbaro & Tacconi [18], illustrates both the feasibility of long-term survival and the risk of complications such as cerebral abscess and permanent visual loss despite adequate surgical treatment. Our third case confirms that even technically successful removal and dural repair may be followed by severe neurological sequelae and medical complications.

In selected cases, intraoperative adjuncts have been reported to further enhance the safety of spear extraction. Endoscopic assistance has been used to directly visualise the barb within the nasal cavity, paranasal sinuses or skull base, allowing precise localisation and controlled dismantling of the tip prior to removal, particularly in complex sinonasal or skull-base trajectories [21]. Similarly, advanced imaging techniques may facilitate accurate identification of the spear trajectory and its relationship to critical neurovascular structures. Although experience with these technologies remains limited to isolated reports, their use may reduce blind manipulation, minimise additional tissue damage and improve surgical precision, especially in anatomically constrained regions.

### Skull-base reconstruction, infection control and complications

Watertight dural closure and skull-base reconstruction are key to preventing CSF leakage, meningitis and intracranial abscess [7–10, 15–17] Various techniques have been reported, including autologous fascial or pericranial grafts, dural substitutes, fibrin glue and endoscopic pedicled flaps for anterior skull-base defects [8, 9, 15]. Bakhos et al. [9] used an endoscopic endonasal approach with a middle turbinate flap to reconstruct an ethmoid roof defect after submental speargun injury, successfully preventing persistent CSF rhinorrhoea. Abarca-Olivas et al. [8] and Solou et al. [10] reported watertight dural closure with dural substitutes and favourable outcomes without infectious complications.

Our second and third patients underwent dural closure with dural substitute patches reinforced with fibrin glue; the bone operculum was not repositioned in Case 3 because of contamination and comminution, resulting in a deliberate craniectomy. Despite extensive contamination of the aerodigestive tract and paranasal sinuses, no intracranial infections occurred, likely due to early debridement, watertight closure and broad-spectrum antibiotic coverage.

Complications reported in the literature include meningitis, brain abscess, CSF leaks, vascular injuries, post-traumatic epilepsy, hydrocephalus and long-term neurological deficits [6–8, 11, 13, 15–17]. Visual loss is relatively frequent in orbitocranial trajectories involving the optic nerve or globe [2, 12, 15, 16]. Cervical cord injury with tetraparesis has been reported when the spear crosses the spinal canal at the upper cervical level [12, 14]. In our series, Case 2 had a remarkably benign neurological course with no residual deficit despite a long frontal trajectory, while Case 3 developed severe right cerebellar syndrome and post-haemorrhagic hydrocephalus, ultimately surviving the acute phase but dying later from pulmonary complications after prolonged immobilisation and critical illness. This delayed mortality parallels other reports in which the initial neurosurgical problem is controlled but systemic complications determine outcome [11, 13].

### Outcomes and role of the maxillofacial surgeon

Overall mortality in published craniofacial speargun injuries remains difficult to estimate because of the small number of cases and reporting bias, but both fatal and favourable outcomes are documented [5–8, 11, 13, 17, 18]. In the series obtained from our review, mortality was observed in 3/27 cases (11.1%) and increased to 3/15 cases (20.0%) when only injuries with confirmed intracranial penetration were considered. Among cases with orbital or orbitonasal trajectories (9/27, 33.3%), visual impairment was reported in 3/9 cases (33.3%), while permanent blindness occurred in 1/9 cases (11.1%). Persistent neurological sequelae following intracranial injury were infrequently reported and were generally mild in patients surviving the acute phase [5–8, 12, 15–17, 22]; in contrast, fatal outcomes were associated with massive intracranial haemorrhage and/or refractory cerebral oedema [11, 13].

Our experience reinforces the impression that early resuscitation, appropriate airway management, comprehensive imaging (including vascular studies when indicated), and meticulous, multidisciplinary surgical planning are critical determinants of outcome. From the perspective of oral and maxillofacial surgery, these cases emphasise several practical points:

- First, even apparently limited facial speargun injuries may conceal complex trajectories; cross-sectional imaging should never be omitted[1-4,6].
- Second, the maxillofacial surgeon often plays a central role in airway management, exposure of facial entry points, control of the barb in extracranial segments and closure of oral and facial wounds, while coordinating with neurosurgery and ENT for skull-base and intracranial components [1-4,7-10,15-17]
- Third, decisions regarding anterograde versus retrograde extraction should be based on the location of the barb, the structures traversed and the feasibility of controlled, direct-vision removal, rather than on convenience alone [1-4,7-12,15-18]

## Supplementary information

Below is the link to the electronic supplementary material.

Supplementary Material 1

Supplementary Material 2 Case 2 – anterograde removal of the speargun

## Data Availability

No datasets were generated or analysed during the current study.

## Declarations

### Clinical trial number

N/A
