# Supplementary material for: Craniofacial speargun injuries: report of three cases, literature review and proposed management guidelines for maxillo-facial surgeons
Source: Oral Maxillofac Surg. 2026 Mar 11;30(1):51. doi: 10.1007/s10006-026-01534-8 (PMC12975845; doi:10.1007/s10006-026-01534-8)
Supplement: Supplementary file 1 — Supplementary Material 1 [file 10006_2026_1534_MOESM1_ESM.docx]

| **Supplementary Table 1. Summary of reported head and neck speargun injuries in the literature.** | | | | | | | | | |
| --- | --- | --- | --- | --- | --- | --- | --- | --- | --- |
| **Author, year** | **Gender, age** | **Etiology** | **Entry point** | **Spear course** | **Brain involvement** | **Radiology** | **Therapy** | **Complication** | **Sequelae** |
| **Chadduck, 1969** | F, 6 | Accident | Right frontal region | Penetrated right frontal bone into frontal lobe | Yes – frontal lobe injury and traumatic cerebral aneurysm | Skull X-ray, cerebral angiography | Craniotomy with removal of spear and hematoma; later aneurysm excision / clipping | Intracerebral hemorrhage, traumatic aneurysm, early seizures | No lasting neurological deficit reported at follow-up |
| **Gutiérrez et al., 1983** | M, 29 | Accident (harpoon discharged while loading) | Left side of nose and medial orbital rim | Through roof of left orbit towards calvaria | Yes – cortical contusion of frontal lobe, mild right upper limb deficit | Skull X-rays | Harpoon cut externally; small left parietal craniectomy, removal along trajectory, dural repair | Immediate right upper limb paresis | Slight distal right hand weakness at 1 month; almost complete recovery by 6 months |
| **Tunbay, 1983** | M, 35 | Accident during spearfishing | Submandibular / upper neck | Through soft tissues to posterior elements of mid-cervical spine | No brain; cervical spinal cord contusion | X-rays, myelography | Cervical laminectomy and spear removal | Acute tetraparesis | Persistent spastic paraparesis; incomplete functional recovery |
| **Hefer et al., 1996 – Case 1** | M, adult (age NR) | Accident | Frontal bone / supraorbital region | Oblique trajectory across anterior cranial fossa toward temporal lobe | Yes – deep parenchymal injury, swelling | Skull X-ray, CT | Craniotomy and attempted controlled spear removal | Massive brain edema, uncontrollable intracranial hypertension | Death (post-operative) |
| **Hefer et al., 1996 – Case 2** | M, adult (age NR) | Accident during underwater fishing | Face (trident impacting maxilla, nose and right orbit) | One prong into left maxillary sinus, one nasal cavity, one through right orbit and orbital roof into frontal lobe | Yes – limited frontal lobe injury | Skull X-ray, CT | Bicoronal approach, removal of each prong under direct vision, reconstruction | Transient ocular motility disturbance | No major long-term neurological sequelae reported |
| **Sadda, 1996** | M, 19 | Accident (friend fired fish-gun) | Right medial canthus | Posterior–inferior through orbit, pterygopalatine / infratemporal fossae, ending in mastoid region | No direct parenchymal penetration; skull base and CSF pathways involved | Skull X-rays, CT | Wide facial and mastoid exposure; spear removal under direct vision; dural repair; antibiotics | CSF otorrhea, facial palsy, optic nerve damage | Permanent blindness and enophthalmos of right eye; facial palsy course NR |
| **Alper et al., 1997 – Case 1** | M, 14 | Accident while loading speargun | Upper lip | Across maxilla toward middle cranial fossa; tip at skull base without intracranial penetration | No | Skull X-rays, CT | Combined intraoral/facial approach with controlled removal; antibiotics | None reported | No sequelae reported |
| **Alper et al., 1997 – Case 2** | M, 23 | Accident | Submandibular region | Superiorly into nasopharynx, close to C1 and skull base | No | Skull X-ray, CT | Transcervical approach; controlled spear extraction | None reported | No sequelae reported |
| **López et al., 2000** | F, 6 | Accident (playing with harpoon gun) | Right frontal region | Oblique perforating trajectory to right occipital bone through right hemisphere | Yes – long intraparenchymal trajectory | CT (no major hemorrhagic lesions reported) | Occipital craniectomy; anterograde removal along trajectory; debridement | None described intra-op | Mild right arm paresis at follow-up |
| **Ribeiro et al., 2009** | M, 9 | Accident (friend fired two-piece fishing harpoon) | Right temporal and orbital region | Two fragments: one through orbit to maxillary sinus; one lodged in temporal soft tissues | No | CT with 3-D reconstruction | Removal of both fragments via small temporal and intraoral incisions; ophthalmologic care; antibiotics | None significant | Minimal cosmetic/functional sequelae only |
| **Ban et al., 2008** | M, 45 | Suicide attempt | Oral cavity / oropharynx | Through clivus into posterior fossa with tip in left occipital bone | Yes – posterior fossa / brainstem–cerebellar involvement | CT, CT-angiography (no vascular lesion) | Occipital craniectomy; anterograde spear removal; debridement; dural closure | None intra-operatively | Facial palsy and cerebellar deficits at discharge; long-term outcome NR |
| **Burnham et al., 2010** | M, 43 | Accident (neighbor discharged harpoon gun) | Medial to left eye | Through ethmoid & maxillary sinuses, pterygoid plate, clivus, posterior arch of C1, abutting spinal canal | No brain; cervical canal violation with cord edema | CT, angiography, postoperative MRI | Controlled extraction under direct vision; immobilization; medical management | Transient spinal cord edema | No permanent neurological deficit |
| **Abarca-Olivas et al., 2011** | M, 34 | Suicide attempt | Submental region | Through tongue, hard palate, right ethmoid, medial orbital apex, anterior cranial fossa, right frontal lobe; exit at right coronal suture | Yes – frontal lobe | CT (no pre-op hemorrhage/edema) | Small craniotomy surrounding exit; anterograde extraction with bone flap; duraplasty; antibiotics & antiepileptics | Transient diplopia | No neurological deficit at discharge; diplopia resolved; no seizures on follow-up |
| **Júnior et al., 2012** | M, 32 | NR | Occipital region near midline | Deep occipital intracranial trajectory with surrounding cerebral edema | Yes | Cranial CT scan | Emergency occipital craniotomy and careful removal; postoperative ICU monitoring | None reported | None |
| **Williams et al., 2014** | M, 55 | Suicide attempt | Lower face / submandibular–oral region (exact entry wording NR) | Upward through skull base into intracranial compartment (details NR in snippet) | Yes – penetrating brain injury | CT, CT-angiography, DSA | Multidisciplinary surgery with craniotomy and controlled removal | NR | Survived; detailed long-term neurological outcome NR |
| **Bakhos et al., 2015** | M, 35 | Suicide attempt | Submental region | Upward into cranial cavity | Yes | CT, CT-angiography | Manual traction of spear followed by endoscopic approach to treat bleeding | None reported | No long-term neurological deficit reported |
| **Brown et al., 2017** | M, 32 | Assault | Right face, 5 cm anterior to right tragus and 2 cm above mid zygomatic arch | Infratemporal region → posterior oropharyngeal wall (right superolateral to left inferomedial, posterior to left tonsil) → exit through left side of neck at level of hyoid; course posterior to ICA/ECA with injury to internal jugular vein | No | CT angiography | Surgical exploration with controlled removal through neck; ligation of internal jugular vein; primary repair of oropharyngeal injury | None reported | None |
| **Barranco et al., 2020** | M, 59 | Suicide | Mouth | Through oral cavity, hard palate, nasal cavity, pterygoid process, sphenoid, orbital apex and optic canal; across entire left cerebral hemisphere to upper parietal bone | Yes – extensive left fronto-parieto-temporal damage, massive hematoma | Brain CT with 3-D reconstruction | Retrograde removal; neurosurgical decompression / craniotomy. | Massive encephalic bleeding during surgery | Death after 2 days from traumatic cranio-encephalic lesions |
| **Macedo Costa et al., 2020** | M, 38 | Accident (homemade spear) | Mandibular symphysis | Through mandible into sublingual space; no intracranial extension | No | CT | Manual spear extraction under GA; open reduction and internal fixation of mandibular fracture | None | Full recovery; no sequelae |
| **Takahashi et al., 2020** | M, 9 | Accidental fall while holding a fishing spear | Left side of the head | Subcutaneous impalement without skull penetration | No | Skull X-ray | Sedation and careful extraction after wound enlargement in emergency setting | None | None |
| **Oearsakul et al., 2021** | M, 26 | Suicide attempt | Mouth / oropharynx | Posterior trajectory to cerebellum; flapper lodged at skull base (infratentorial injury) | Yes – cerebellar penetrating injury | CT, CT-angiography | Combined transoral and suboccipital approach; retrograde spear removal (flapper at skull base); dural repair | None significant | No permanent neurological deficit at follow-up |
| **Lacuata et al., 2021** | M, 22 | Intentional harpoon gun injury (shot while sleeping) | Left preauricular region | Oblique trajectory across posterior maxilla, sphenoid sinus, and bilateral posterior ethmoid sinuses; tip lodged at right orbital apex | No | Craniofacial CT scan and skull radiographs | Combined external and endoscopic transnasal approach with diamond burr to dismantle hook and controlled extraction | None reported | None |
| **Öncül et al., 2023** | M, 24 | Accidental speargun injury | Bilateral submandibular regions | Penetration through floor of mouth with bilateral tongue lacerations and hard palate perforation; extension into nasal cavity and frontal sinus | No | Maxillofacial CT scan | Emergency surgery with tracheotomy; bilateral harpoon removal (retrograde on one side, cervical exploration on the other); primary repair | None reported | None |
| **Solou et al., 2025** | M, 38 | Suicide attempt | Submandibular area | Through oral cavity and skull base into frontal lobe with frontal exit | Yes – frontal lobe traumatic brain injury | CT, CT-angiography | Craniotomy with anterograde spear removal and debridement; antibiotics and antiepileptics | None reported | Favourable neurological outcome; no major persistent deficit reported |
